# Identification and differential analysis of noncoding RNAs in response to drought in *Phyllostachys aureosulcata* f. *spectabilis*

**DOI:** 10.3389/fpls.2022.1040470

**Published:** 2022-11-10

**Authors:** Yang Yang, Yuanmeng Gao, Yiqian Li, Xueping Li

**Affiliations:** International Center for Bamboo and Rattan, Key Laboratory of National Forestry and Grassland Administration/Beijing for Bamboo and Rattan Science and Technology, Beijing, China

**Keywords:** ncRNAs, drought stresses, GO enrichment, KEGG enrichment, miRNA decoys

## Abstract

The role of noncoding RNAs (ncRNAs) in plant resistance to abiotic stresses is increasingly being discovered. Drought stress is one of the most common stresses that affecting plant growth, and high intensity drought has a significant impact on the normal growth of plants. In this study, a high-throughput sequencing was performed on plant tissue samples of *Phyllostachys aureosulcata* f. *spectabilis* C. D. Chu et C. S. Chao by drought treatment for 0, 2, 4 and 6 days. The sequencing results were analysed bioinformatically. We detected 336,946 RNAs among all 12 samples, including 192,098 message RNAs (mRNAs), 142,761 long noncoding RNAs (lncRNAs), 1,670 circular RNAs (circRNAs), and 417 microRNAs (miRNAs). We detected 2,419 differentially expressed (DE) ncRNAs, including 213 DE circRNAs, 2,088 DE lncRNAs and 118 DE miRNAs. Then, we used Gene Ontology (GO) and Kyoto Encyclopedia of Genes and Genomes (KEGG) to functionally predict DE ncRNAs. The results showed that most DE ncRNAs are involved in the response to drought stress, mainly in biochemical reactions involved in some metabolites, as well as in organelle activities. In addition, we validated two random circRNAs and demonstrated their circularity. We also found a stable internal reference gene available for *Phyllostachys aureosulcata* f. *spectabilis* and validated the accuracy of this experiment by quantitative real-time polymerase chain reaction (qRT-PCR).

## Introduction

*Phyllostachys aureosulcata* f. *spectabilis* C. D. Chu et C. S. Chao (*P. aureosulcata*) is a cultivated variant of *Phyllostachys aureosulcata* (Li et al., 2017). This kind of bamboo characterized by golden yellow poles, naturally long turquoise stripes. *P. aureosulcata* can stay green for all seasons, so it is especially suitable for cultivation under natural conditions in most areas (Zhang, 2014). According to the reasons above, the plant has become a rare bamboo treasure for landscaping and gardening worldwide. Especially after the Beijing Olympic Games, *P. aureosulcata* has been loved by people all over the world with its same name as the gold medal of the 2008 Beijing Summer Olympics and large area application in Olympic Park (Song et al., 2021b).

Drought is one of the environmental stresses that has the greatest worldwide impact on agricultural production (Kumar et al., 2018). In recent years, due to the drastic changes in global climate, the drought stresses encountered by plants under natural conditions have gradually increased in number and magnitude. Severe drought stress can cause physical damage and physiological or biochemical disorders, which in turn can alter plant morphology (Hussain et al., 2018), and this can be lethal to plants. Drought stress may also affect a range of physiological and biochemical reactions, including photosynthesis, chlorophyll synthesis, nutrient metabolism, ion uptake and transfer, respiratory action, and carbohydrate metabolism, inhibiting plant growth, and thus adversely affecting plant growth and yield (Li et al., 2011; Patel et al., 2020).

The plant response to drought stress is a complex biological process that involves a common dynamic change in metabolite composition and gene expression (Urano et al., 2009). Drought stress directly affects primary metabolism and leads to changes in the biosynthesis and transport of primary and secondary metabolites in plants (Ramakrishna and Ravishankar, 2011; Ma et al., 2020). At the gene expression level, plants have evolved various cascaded signalling networks that are used to regulate drought-responsive genes to produce various types of proteins, including transcription factors, enzymes, molecular chaperones, and other functional proteins, and ultimately achieve drought tolerance in plants (Hu and Xiong, 2014; Kumar et al., 2017; Kumar et al., 2021). Drought-responsive genes are involved in signalling cascades, transcriptional regulation, such as transcription factors and protein kinases/phosphatases, and the expression of functional proteins that protect cell membranes (Nakashima et al., 2014; Jogawat et al., 2021).

Noncoding RNAs (ncRNAs) are ubiquitous in plants, although for a long time, ncRNAs were viewed as pointless silent regions; later on, an increasing number of studies demonstrated that ncRNAs have a variety of regulatory effects (Bushati and Cohen, 2007; Bartel, 2009; Mercer et al., 2009). Identification and analysis in 15 diverse flowering plant species showed that 44% of ncRNAs have significant similarity to benchmark protein-coding or RNA genes and therefore have a high probability of being part of functional genes (Lloyd et al., 2018).

There are many types of ncRNAs, the main ones are microRNAs (miRNAs), long ncRNAs (lncRNAs), and circular RNAs (circRNAs), which usually interact with each other and commonly regulate the expression of target genes (Song et al., 2021a). ncRNAs have multiple functions. For example, ncRNAs are key regulators in several developmental processes such as leaf morphogenesis, vegetative phase change, flowering time and response to environmental cues, and the ncRNAs regulate the expression of abiotic stresses, such as drought, salt and high temperature, in plants facing different biotic stresses, salt, high temperature and other abiotic stresses, participation in plant immunity, and regulation in response to macronutrient stress in plants (Millar, 2020; Tahir Ul Qamar et al., 2020; Jin et al., 2021; Li et al., 2021).

In *Bambusoideae*, studies on ncRNAs have also made some progress. For example, they regulate the nutrients necessary for growth, influence the development of tissue and organs, and respond to abiotic stresses (Wang et al., 2016; Cheng et al., 2020; Wang et al., 2021), but no systematic studies about ncRNAs have been carried out in *P. aureosulcata*. Therefore, in this study, we systematically counted all types of ncRNAs in *P. aureosulcata* from the most common abiotic impact factor in growth: drought stress, focusing on ncRNAs involved in the stress response to drought stress, and performed KEGG/GO enrichment analysis. A stable reference gene was screened for *P. aureosulcata* and then verified by real-time quantitative PCR (qRT-PCR) using circRNAs as the point of penetration.

## Materials and methods

### Plant materials

*Phyllostachys aureosulcata* f. *spectabilis* C. D. Chu et C. S. Chao (*P. aureosulcata*) were grown in Dinghuo town, Yangzhou city, Jiangsu Province, China (119°38′10″E, 32°29′15″N). The average annual temperature is 14.8-15.3°C, and the light time is approximately 1896-2182 h per year. In addition, the annual precipitation can reach 1,048 mm, which is suitable for *P. aureosulcata* to grow. After digging the bamboo out of the soil, they were transplanted into planting pots (40 cm × 50 cm). After the seedlings were lowered, potted seedlings with good growth conditions were selected and treated with natural drought stress and divided into 4 groups with 3 replicates in each group. The drought treatment gradient was 0 days, 2 days, 4 days and 6 days. Leaves were collected from the same part of different plants, frozen with liquid nitrogen and saved at -80°C until further use. The soil water content is as follows: 0 day of drought treatment: 36.83%, 2 days of drought treatment: 29.22%, 4 days of drought treatment: 25.37%, 6 days of drought treatment: 22.15%.

### RNA extraction, cDNA library construction and RNA sequencing

After dehydration with liquid nitrogen, the plant tissues were ground into powder, and total RNA was extracted using CLB, CTAB, and the RN40-EASYspin Plant RNA Rapid Extraction Kit from Aidlab Biotechnologies Co., Ltd., Beijing, China. Then, a Nanodrop 2000 (Thermo Fisher Scientific, Waltham, MA, USA) was used for concentration testing, and an Agilent 2100 Bioanalyzer (Agilent, Santa Clara, CA, USA) and LabChip GX Touch Nucleic Acid Analyser (PerkinElmer, Waltham, MA, USA) were used for integrity testing.

For cDNA library construction, a Ribo-off rRNA Depletion Kit (Plant) N409-02 was used. First, rRNA was removed from total RNA with rRNA probes and ferrite beads. Second, the interruption mixture was added to the library system reserved in step one to interrupt the rRNA-depleted RNA. Third, we synthesized the first strand of cDNA, synthesized the second chain according to the first chain and purified it. Fourth, end-point repair and dA-tailing of the above products were performed. Finally, reverse transcription was used to synthesize cDNA, followed by PCR amplification, polyacrylamide gel electrophoresis (PAGE) to separate the target DNA fragments, and cut gel recovery to obtain the cDNA library. While constructing the circRNA-seq libraries, add a step that removes the linear RNA with RNase R before the last step.

The quality of the constructed cDNA libraries was checked by the Qsep-400 method. The libraries that met the requirements were sequenced using Illumina NovaSeq 6000. The sequencing platform was an Illumina NovaSeq 6000 platform (San Diego, CA, USA), and the sequencing reagent was a NovaSeq 6000 S4 Reagent Kit (San Diego, CA, USA).

To identify the lncRNAs, three computational approaches including CPC2/CNCI/Pfam/CPAT were combined to sort non-protein coding RNA candidates from putative protein-coding RNAs in the unknown transcripts. Putative protein-coding RNAs were filtered out using a minimum length and exon number threshold. Transcripts with lengths more than 200 nt and have more than two exons been selected as lncRNA candidates (Li et al., 2015). To identify circRNAs, use CIRI software to compare with the reference gene sequences, generate SAM files, and scan PCC signals (paired chiastic clipping signals) from the SAM files for the CIGAR values analyzed in the SAM files. The CIGAR values in the junction read are characterized by xS/HyM or xMyS/H (Gao et al., 2015). Besides, to identify the sRNA. Raw reads of fastq format were firstly processed through in-house perl scripts. In this step, clean reads were obtained by removing reads containing adapter, reads containing ploy-N and low-quality reads from raw data. And reads were trimmed and cleaned by removing the sequences smaller than 18 nt or longer than 30 nt (Friedländer et al., 2012; Zhang et al., 2015).

### Selection of internal reference genes and validation of circRNAs in *P. aureosulcata*

Moso bamboo (*Phyllostachys edulis*) is the most closely related and well-studied bamboo species of *P. aureosulcata*, so 11 candidate genes were selected for use as reference genes in moso bamboo (Fan et al., 2013; Qi et al., 2013; Wu et al., 2018). The conserved sequences were downloaded from the National Center for Biotechnology Information (NCBI) database (http://www.ncbi.nlm.nih.gov), and then Primer Premier 5 software was used to design primers for cloning (**Supplementary Table 2**). The annealing temperature of each primer was approximately 62°C, and the length was approximately 20-26 bp. Additionally, the length of each amplification product was between 100 bp and 180 bp. Respectively plucking the *P. aureosulcata* roots, stems, shoots, and leaves from different drought treatments in the *Plant materials* section (natural drought for 0 d, 2 d, 4 d, and 6 d), each tissue sample was obtained from at least three plants under good growth conditions. Different RNA was extracted by the TRIzol method (Total RNA Extraction Kit, TianMo Biotech, Tianjin, China), and 1 ng of each RNA was reverse transcribed to obtain cDNA for relative expression analysis of target genes by qRT-PCR. The total RNA was taken and directly reverse transcribed using PrimeScript RT Master Mix (Takara, Beijing, China) to obtain gDNA. Then, the total RNA was delinearized using Ribonuclease R (RNase R) and reverse transcribed using PrimeScript RT Master Mix to obtain cDNA with the linear RNA removed. A Nanodrop 2000 (Thermo Fisher Scientific) was used to check the RNA quality to ensure that the A260/A280 ratio of the RNA samples was between 1.9 and 2.1.

PCR amplification was carried out using cDNA as a template. The PCR products were visualized by agarose gel electrophoresis, and genes with clear and unique bands without primer-dimer formation were selected and confirmed by Sanger sequencing. After confirming that the sequences were correct, qRT-PCR was performed using 2×SYBR qPCR MasterMix (Real-Times Biotechnology, Beijing, China) on a Roche LightCycler 480 fluorescence quantification instrument (Basel, Switzerland). The qRT-PCR procedure was as follows: 95°C for 1 min, followed by 40 cycles of 95°C for 15 s and 60°C for 60 s. After obtaining the data, the results were imported into NormFinder software for statistical analysis, and the gene expression stability value (S value) was calculated (Andersen et al., 2004). The gene with the smallest S value was obtained as the most suitable internal reference gene for *P. aureosulcata* under the present experimental conditions.

The circRNAs were randomly selected, and the convergent and divergent primers were designed using Primer Premier 5. Then, cDNA and gDNA were used as templates with two sets of primers (**Supplementary Table 2**). The PCR products were also visualized by agarose gel electrophoresis and confirmed by Sanger sequencing. Using the system and apparatus above, qRT-PCR was performed with the selected PP2A as the reference gene, and the relative expression of circRNAs was calculated using the 2^-ΔΔCt^ method (Livak and Schmittgen, 2001).

### Differential expression analysis

The DESeq R package (1.10.1) was used for differential expression analysis. DESeq provides statistical routines for determining differential expression in digital gene expression data using a model based on the negative binomial distribution. The resulting *P* values were adjusted using Benjamini and Hochberg’s approach for controlling the false discovery rate. Genes with an adjusted *P* value <0.01 and absolute value of log_2_(fold change) >1 found by DESeq were considered differentially expressed.

### Functional prediction of DE RNAs

Gene Ontology (GO) enrichment analysis of the differentially expressed genes (DEGs) was implemented by the topGO R package. In addition, we used KEGG Orthology Based Annotation System (KOBAS) software to test the statistical enrichment of differentially expressed genes in KEGG pathways. Benjamini and Hochberg’s methods were used to adjust *P* values. In the detection of DE circRNAs, fold change greater than or equal to 2 and *P* values less than 0.05 were selected as screening criteria. The mRNA-miRNA-circRNA-lncRNA network was generated by Cytoscape (v3.7.2) (Shannon et al., 2003) and target gene prediction with TargetFinder software (Bo and Wang, 2005).

## Results

### Identification and characterization

To identify and associate ncRNAs with drought in *P. aureosulcata*, samples were taken from *P. aureosulcata* at 0 (P1), 2 (P2), 4 (P3) and 6 (P4) days of drought treatment, and RNA-Seq libraries were constructed, which were subjected to sequencing using the Illumina HiSeq 2500 platform. A total of 230.90 Gb of clean data was obtained from 12 samples, the percentage of Q30 bases in each sample was not less than 93.89%, and the guanine and cytosine (GC) content was approximately 44.58% (**Supplementary Table 1**).

**Figure 1:**
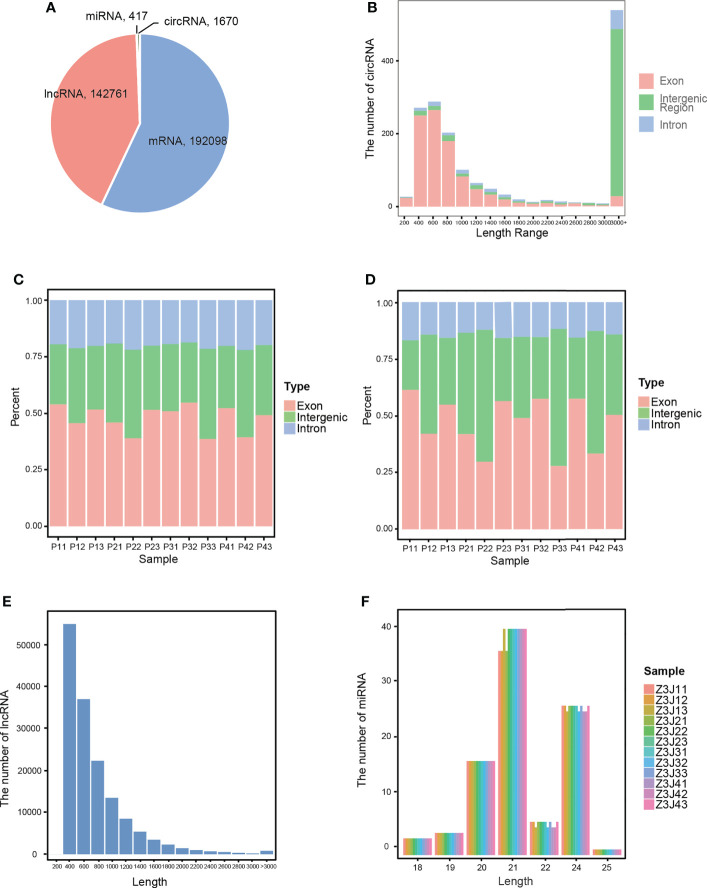
Characterization of RNAs. **(A)** The number of each type of RNA in all samples. **(B)** The length distribution of circRNAs. **(C)** The type and percentage of circRNAs in each sample. **(D)** The type and percentage of lncRNAs in each sample. **(E)** The length distribution of lncRNAs. **(F)** The length distribution of miRNAs in each sample. Drought treatment with three biological repetitions each: 0 day (P11, P12, P13), 2 days of drought treatment (P21, P22, P23), 4 days of drought treatment (P31, P32, P33) and 6 days of drought treatment (P41, P42, P43).

Using the FASTQ data obtained from high-throughput sequencing, several RNAs were predicted and identified separately, and the quantities of several ncRNAs were counted in combination with known RNAs. The results are shown in **Figure 1A**. We detected 336,946 RNAs among all 12 samples, including 192,098 mRNAs, 142,761 lncRNAs, 1,670 circRNAs, and 417 miRNAs. Then, we used 3 different software programs to analyse different kinds of ncRNAs to obtain more details. CircRNAs were predicted using circRNA identifier (CIRI) (Gao et al., 2015), and the distribution statistics of the length of predicted circRNAs were carried out. As shown in **Figure 1B**, most of the circRNAs were between 200-800 bp in length. The type statistics of circRNAs performed in **Figure 1C** also showed that most of the circRNAs were classified as exon circRNAs. Then, using HISAT2 (Li et al., 2015) to predict lncRNAs. The predicted lncRNAs were distributed with length and class statistics, as shown in **Figures 1D, E**. Most of the lncRNAs were between 400-800 bp in length and were classified as exon lncRNAs. Finally, using miRDeep2 software, adjusting and changing its parameters and scoring system to make it suitable for the prediction of plant miRNAs (Friedländer et al., 2012; Zhang et al., 2015). As shown in **Figure 1F**, most of the miRNAs were 21 or 24 bp in length.

### Differentially expressed ncRNAs under drought treatment

**Figure 2:**
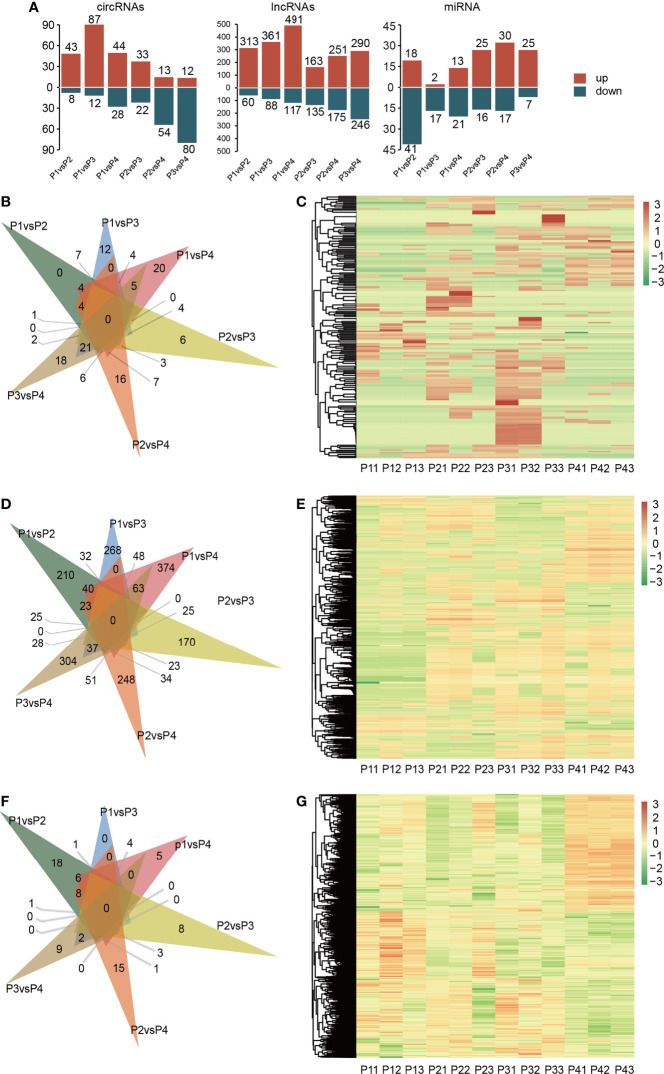
Differential expression analysis of ncRNAs in response to drought stress treatment. Drought treatment with three biological repetitions each: 0 day (P11, P12, P13), 2 days of drought treatment (P21, P22, P23), 4 days of drought treatment (P31, P32, P33) and 6 days of drought treatment (P41, P42, P43). **(A)** The regulation of different kinds of DERNA between each treatment. **(B)** Venn diagram of DE circRNAs at six group of treatment. **(C)** Heatmap of expression of all DE circRNAs in all samples, the horizontal coordinates in the diagram represent the sample names and the clustering results of the samples, the vertical coordinates represent the differential genes and the clustering results of the genes. Different columns in the graph represent different samples, and different rows represent different genes. The color represents the expression level of the gene in the sample log10(FPKM+0.000001). **(D)** Venn diagram of DE circRNAs at six group of treatment. **(E)** Heatmap of expression of all DE lncRNAs in all samples. **(F)** Venn diagram of DE miRNAs at six group of treatment. **(G)** Heatmap of expression of all DE miRNAs in all samples.

A total of 2,419 differentially expressed ncRNAs were detected under different gradients of drought treatment. Among them, a total of 213 DE circRNAs were detected. There were also 2,088 DE lncRNAs. The total number of DE miRNAs with differential epistatic responses was 118 (**Figure 2**). The clustering analysis of differentially expressed lncRNAs, circRNAs and miRNAs was performed, and the results are shown in **Figure 2**.

### Reference gene selection and quantitative real-time polymerase chain reaction

**Figure 3:**
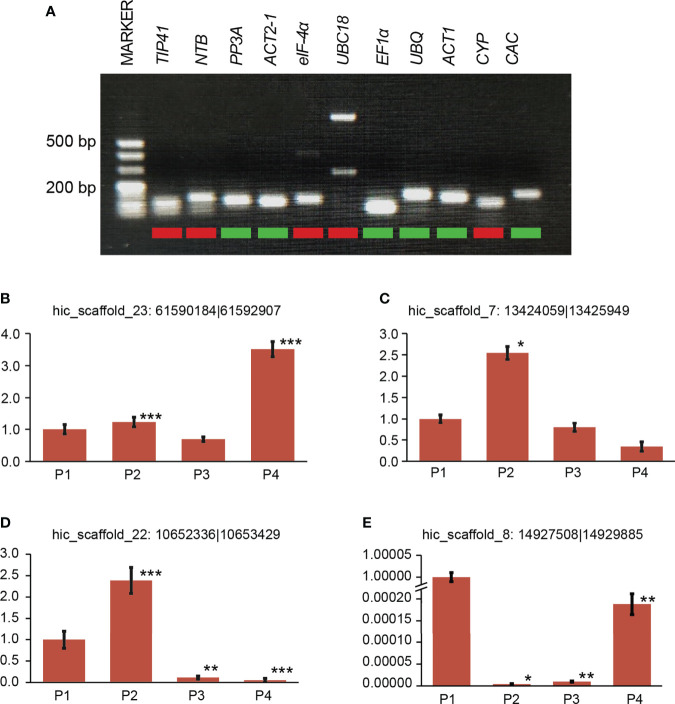
Reference gene selection and qRT-PCR validation of four DE circRNAs **(A)** PCR amplification of 11 reference genes. **(B-E)** Relative expression of four selected DE circRNAs measured by RT-qPCR. For RT-qPCR, three replicates were performed. Asterisks on bars indicate significant differences between different stages using a *t*-test. Error bars represent the standard error of the mean. Drought treatment: 0 day (P1), 2 days of drought treatment (P2), 4 days of drought treatment (P3) and 6 days of drought treatment (P4). * *p* < 0.05, indicates a significant difference. ** *p* < 0.01, indicates a profoundly significant difference. *** *p* < 0.001, indicates a profoundly significant difference.

Among the 11 candidate reference genes, 6 candidate genes were measured by qRT-PCR products of agarose gel electrophoresis and showed a single specific band with the same size of the target fragment and no primer dimer below the band. The six selected reference genes were Phosphoprotein Phosphatase 2A (*PP2A*), Actin2-1 (*ACT2-1*), Elongation Factor 1 Alpha (*EF1α*), Ubiquitin (*UBQ*), Actin-1 (*ACT1*), and Clathrin Adaptor Complexes Medium Subunit (*CAC*) (**Figure 3A**). Their primers were amplified with high efficiency and met the criteria of qRT-PCR experiments for gene expression stability analysis. For the six selected reference genes, qRT-PCR analysis was performed with cDNAs under different drought treatments (0 days, 2 days, 4 days and 6 days) and cDNAs from different plant organs (roots, stems, leaves and shoots) as templates. The results were imported into NormFinder software for statistical analysis (Andersen et al., 2004), and the gene expression stability values (S values) were calculated (**Table 1**). The gene with the smallest S value under both situations was obtained as PP2A, and PP2A was identified as the most suitable internal reference gene for *P. aureosulcata* under the present experimental conditions.

**Table 1:** Ranking order of reference genes determined by NormFinder under drought treatments.

	Ranking order of stability under different conditions	
Gene Name	Different treatments	Different tissues
*PP2A*	1 (0.08)	1(1.24)
*UBQ*	2 (0.12)	2 (1.9)
*EF1α*	3 (0.13)	3 (2.31)
*CAC*	4 (0.15)	5 (3.13)
*ACT2-1*	5 (0.15)	6 (3.16)
*ACT1*	6 (0.17)	4 (3.02)

In addition, to verify the reliability of the gene expression profile, we randomly selected four DE circRNAs for expression analysis (**Figure 3**). qRT-PCR results showed expression patterns consistent with the RNA-seq results.

### Validation of circRNAs

**Figure 4:**
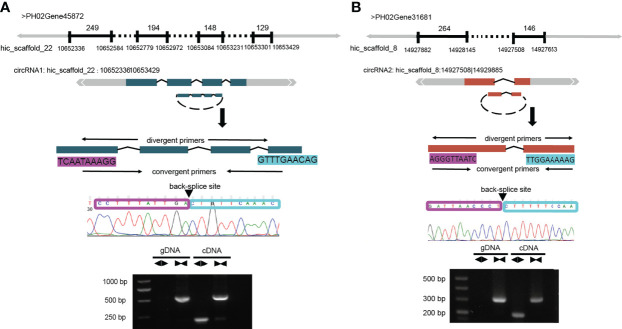
Validation of circRNAs by PCR and Sanger sequencing. **(A)** Schematic representation of the validation of circRNA1: hic_scaffold_22:10652336|10653429. circRNA1 is formed after the circularization of 4 exons in the PH02Gene45872 gene. **(B)** Schematic representation of the validation of circRNA2: hic_scaffold_8:14927508|14929885. circRNA2 is formed after the circularization of 2 exons in the PH02Gene31681 gene. PCR amplification was performed after designing two sets of primers (convergent and divergent) for these two genes; clear bands could be observed in the products by agarose gel electrophoresis (AGE), and the amplified products could be sequenced to obtain the complete sequences at the first and last junctions.

To verify the accuracy of our identified circRNAs, we randomly selected two circRNAs using PCR and Sanger sequencing to validate them and successfully confirmed their circularity. Unlike linear RNAs, circRNAs are a loop, so two sets of primers (convergent and divergent) and two templates (genomic DNA [gDNA] and complementary DNA [cDNA]) were used to verify it (**Figure 4A**). The normal convergent primers can obtain amplification bands in both gDNA and cDNA, while the reverse divergent primers can only obtain amplification bands in cDNA (**Figure 4**).

### Functional prediction of DE ncRNAs by *Gene Ontology* (GO) and *Kyoto Encyclopedia of Genes and Genomes*

**Figure 5:**
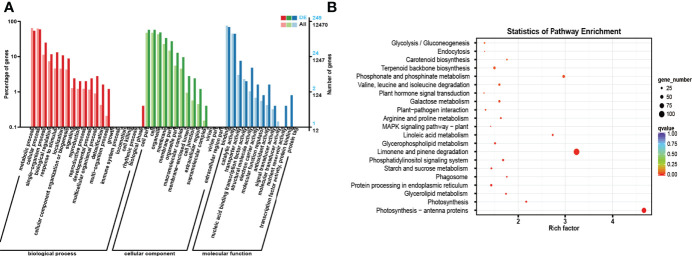
GO and KEGG enrichment analyses of the host genes of all DE RNAs. **(A)** GO enrichment analysis of the host genes of all DE ncRNAs. The horizontal coordinate is the GO classification, the left side of the vertical coordinate is the percentage of the number of genes enriched in that GO term, and the right side is the number of genes. **(B)** KEGG enrichment analysis of the host genes of all DE ncRNAs. Each circle in **Figure 5** represents a KEGG pathway, the vertical coordinate indicates the top 20 pathway names, and the horizontal coordinate is the enrichment factor, which represents the ratio of the proportion of differential genes annotated to a pathway (gene ratio) to the proportion of all genes annotated to that pathway (background ratio). The colour of the circle represents the q value, which is the P value after correction for multiple hypothesis testing, and the size of the circle indicates the number of genes enriched in the pathway.

**Figure 6:**
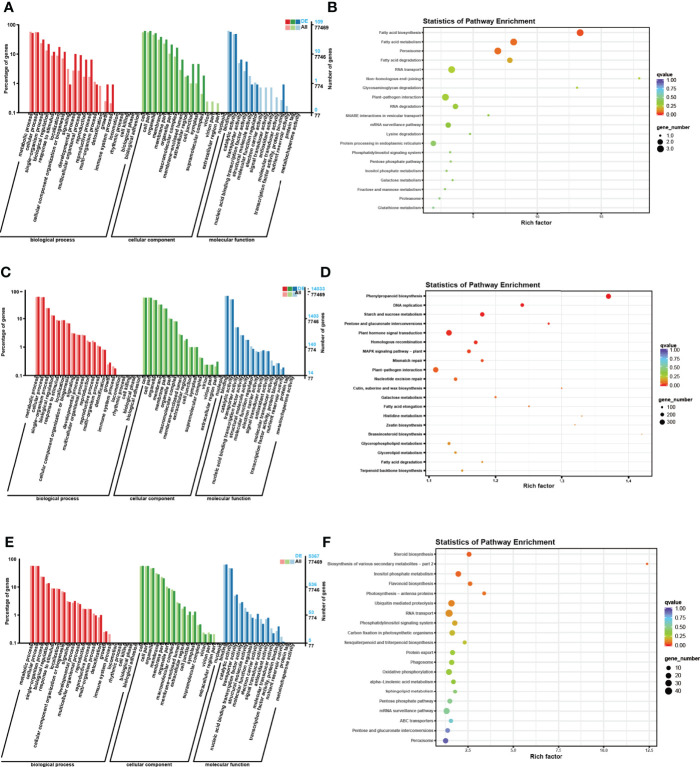
GO and KEGG enrichment analyses of the host genes of DE RNAs separately. **(A)** GO enrichment analysis of the host genes of DE circRNAs. The horizontal coordinate is the GO classification, the left side of the vertical coordinate is the percentage of the number of genes enriched in that GO term, and the right side is the number of genes. **(B)** KEGG enrichment analysis of the host genes of DE circRNAs. Each circle in **Figure 6** represents a KEGG pathway, the vertical coordinate indicates the top 20 pathway names, and the horizontal coordinate is the enrichment factor, which represents the ratio of the proportion of differential genes annotated to a pathway (gene ratio) to the proportion of all genes annotated to that pathway (background ratio). The colour of the circle represents the *q* value, which is the *P* value after correction for multiple hypothesis testing, and the size of the circle indicates the number of genes enriched in the pathway. **(C)** GO enrichment analysis of the host genes of DE lncRNAs. **(D)** KEGG enrichment analysis of the host genes of DE circRNAs. **(E)** GO enrichment analysis of the host genes of DE miRNAs. **(F)** KEGG enrichment analysis of the host genes of DE miRNAs.

To further understand the functions and characteristics of DE ncRNAs, enrichment analyses of all DE ncRNAs, DE circRNAs, DE lncRNAs, and DE miRNAs were performed using the *Gene Ontology* (GO) and *Kyoto Encyclopedia of Genes and Genomes* (KEGG) databases (**Figures 5**, **6** and **Supplementary Tables 3****,** **4**).

According to the results of GO enrichment analysis, the host genes were involved in three categories: biological process (BP), cellular component (CC), and molecular function (MF). In the BP section, most host genes were enriched in metabolic process (GO: 0008152), cellular process (GO: 0044763), single organism process (GO: 0006465) and biological regulation (GO: 0065007). In the CC category, most host genes were enriched in cell (GO: 0005623), cell part (GO: 0044464), organelle (GO: 0043226) and membrane (GO: 0016020). In addition, in the MF category, most host genes were enriched in binding (GO: 0005488), catalytic activity (GO: 0003824), transporter activity (GO: 0005215) and nucleic acid binding transcription factor activity (GO: 0003700).

For the KEGG enrichment analysis, the most closely related metabolic pathways of the host genes of all DE ncRNAs were glycolysis/gluconeogenesis (ko00010), endocytosis (ko04144), Carotenoid biosynthesis (ko00906), Terpenoid backbone biosynthesis (ko00900), Phosphonate and phosphinate metabolism (ko00440). Besides, the results showed that the most closely related metabolic pathways of the host genes of DE circRNAs under drought treatment were fatty acid biosynthesis (ko00061), fatty acid metabolism (ko01212), peroxisome (ko04146), fatty acid degradation (ko00071) and RNA transport (ko03013). In addition, the most closely related metabolic pathways of the host gene of DE lncRNAs are phenylpropanoid biosynthesis (ko00940), DNA replication (ko03030), starch and sucrose metabolism (ko00500), pentose and glucuronate interconversions (ko00040) and plant hormone signal transduction (ko04075). Meanwhile, the most closely related metabolic pathways of the host genes of DE miRNAs are steroid biosynthesis (ko00905), biosynthesis of various secondary metabolites-Part 2 (ko00998), inositol phosphate metabolism (ko00562), flavonoid biosynthesis (ko00941) and photosynthesis-antenna proteins (ko00196).

### Functional prediction of DE ncRNAs based on the mRNA-miRNA-circRNA-lncRNA network

**Figure 7:**
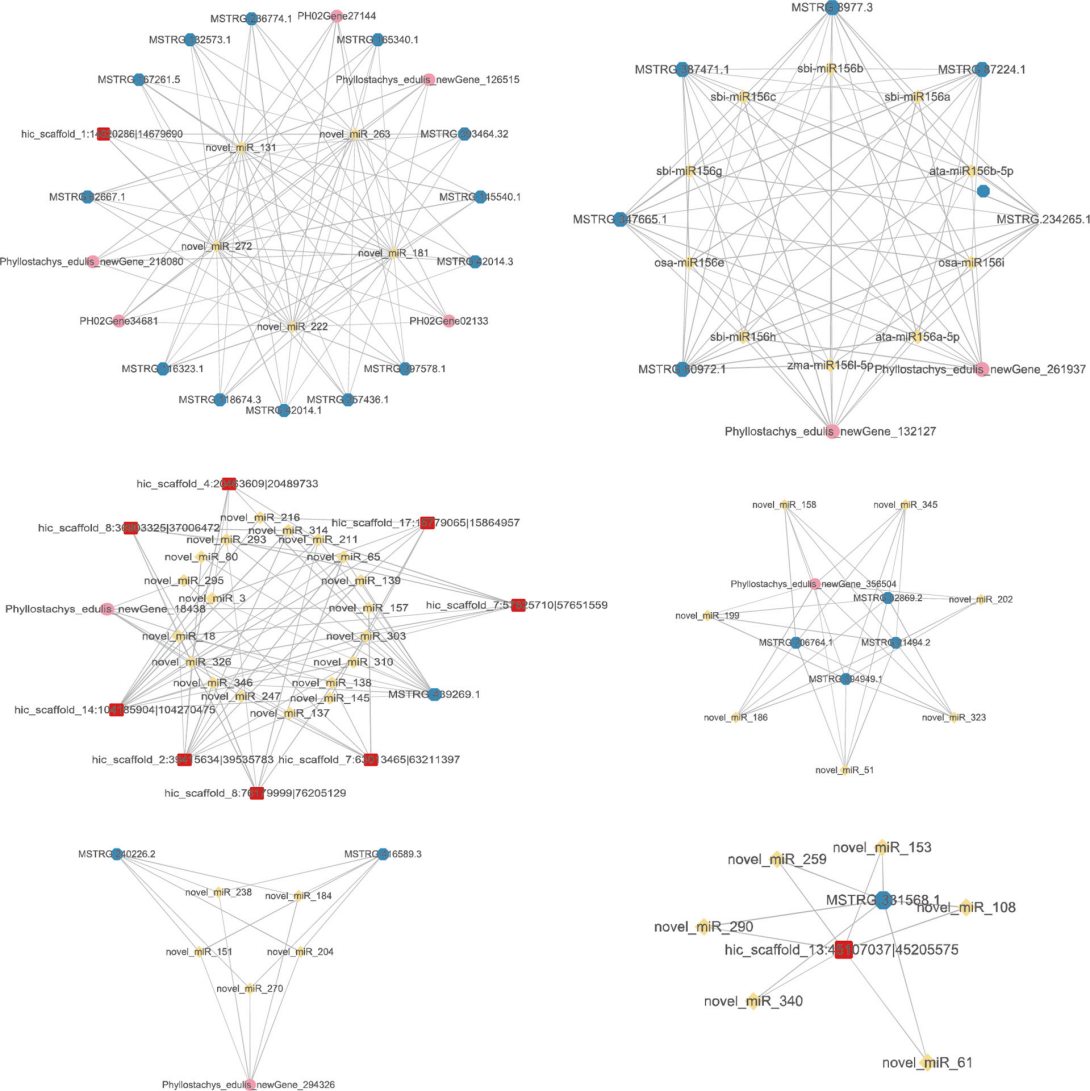
The mRNA-miRNA-circRNA-lncRNA interaction network of P1 treatment with drought treatment P4. Yellow nodes: miRNAs. Red nodes: circRNAs that may be miRNA decoys. Blue nodes: lncRNAs that may be miRNA decoys. Pink nodes: mRNAs that may be miRNA targets.

To further explore the functions of DE ncRNAs, we first predicted DE circRNAs and DE lncRNAs as miRNA decoys and mRNAs as miRNA targets. Then, based on the potential relationship between these RNAs, three mRNA-miRNA-circRNA-lncRNA regulatory networks were constructed by screening the differentially expressed mRNAs (**Figure 7**). The reciprocal relationship networks of P1 treatment with drought treatment P2 (**Supplementary Figure 1**), P3 (**Supplementary Figure 1**) and P4 (**Figure 7**). Meanwhile, the mRNAs involved in the above three mRNA-miRNA-circRNA-lncRNA regulatory networks were annotated and analysed. We found that these mRNAs were mainly annotated to the following GO entries:

Molecular Function: DNA binding (GO: 0003677), nucleic acid binding (GO: 0003676), zinc ion binding (GO: 0008270), RNA binding (GO: 0003723), RNA-directed DNA polymerase activity (GO: 0003964), aspartic-type endopeptidase activity (GO: 0004190), transporter activity (GO: 0005215), phenylalanine ammonia-lyase activity (GO: 0045548). Cellular Component: Nucleus (GO: 0005634), Plastid (GO: 0009536), Integral component of membrane (GO: 0016021), Membrane (GO: 0016020), Biological Process: regulation of transcription, DNA-templated (GO: 0006355), Mitochondrion (GO: 0005739), Cytoplasm (GO: 0005737). Biological Process: DNA integration (GO: 0015074), Regulation of transcription, DNA-templated (GO: 0006355), Auxin-activated signalling pathway (GO: 0009734), RNA-dependent DNA biosynthetic process (GO: 0006278), DNA recombination (GO: 0006310), proteolysis (GO: 0006508), L-phenylalanine catabolic process (GO: 0006559), Cinnamic acid biosynthetic process (GO: 0009800).

Based on the above two chapters, the KEGG/GO enrichment analysis showed that non-coding RNAs are mainly involved in the following biological processes: 1, metabolism of primary/secondary metabolites. This is the class with the highest number of enriched ncRNAs and involving GO/KEGG entries. By regulating the synthesis and metabolism of some metabolites closely related to the daily life activities of plants and key compounds in key biological cycles (e.g., saccharides, alcohols, proteins, lipids, etc.) to counteract the effects of external environmental water changes. 2, Organelle activity. By regulating the activity of organelles such as peroxisome, plastid, and mitochondrion, the strength of relevant biochemical reactions in plants is mobilized to increase/decrease, thus enhancing the resistance of plants to external water changes. 3, hormones. By regulating plant hormone signal transduction to make changes in plant physiological and biochemical metabolism, thus improving drought resistance and reducing plant damage. 4, Increase drought resistance by regulating RNA and DNA synthesis/recombination/translation, etc. 5, Regulate the ions in the tissues. By regulating the activity of cell membrane, as well as regulating the state of ions such as Zn^2+^ and Ca^2+^ in the cell, and regulating the osmotic pressure in the tissues to resist external water changes.

## Discussion

As global warming increases, environmental temperatures are gradually rising, which greatly increases the probability and duration of drought. As a common greenery bamboo, *P. aureosulcata* is planted mostly in urban environments and grows naturally, which further deepens the impact of drought stress on it. Meanwhile, the role of ncRNAs has been gaining attention in recent decades, from inconsequential transcriptional “noise” to “a new continent in the RNA world”. ncRNAs are mainly classified into long noncoding RNAs (lncRNAs), which are longer than 200 nt, and small noncoding RNAs (sRNAs), which are smaller than 200 nt (Gelaw and Sanan-Mishra, 2021). Many experimental reports have shown that ncRNAs and their target genes play important roles in plant development, environmental responses, and biotic and abiotic stress responses (Ohtani, 2017).

With the advancement of high-throughput sequencing technology, many new ncRNA transcripts have been identified in different species (Di et al., 2014; Wang et al., 2015; Deng et al., 2018; Yuan et al., 2018). Bioinformatics analysis has proven to be an important tool for exploring differences in plant responses to drought stress and has been applied to several species, including *Arabidopsis* (Qin et al., 2017), rice (Mutum et al., 2016), maize (Zhang et al., 2014), tomato (Zhong et al., 2013), sorghum (Fracasso et al., 2016), and coffee (Mofatto et al., 2016), and many drought-related DE ncRNAs have been identified. These studies revealed the complexity of the regulation between drought stress and ncRNAs and revealed that ncRNAs play important roles in many important biological processes. Therefore, understanding the regulatory mechanisms of ncRNAs in response to drought will provide a molecular basis for plant resistance studies. However, genomic identification and characterization of known and novel ncRNAs under drought stress of *P. aureosulcata* are still lacking. Therefore, in this research, we conducted an overall statistical analysis, identification and analysis of all species of DE ncRNAs in *P. aureosulcata* under drought treatment and focused on analysing circRNAs in addition to counting conventional lncRNAs and miRNAs. A total of 2,419 differentially expressed ncRNAs were detected under different gradients of drought treatment, including 213 DE circRNAs, 2,088 DE lncRNAs and 118 DE miRNAs. Among these DE ncRNAs, the number of upregulated versus downregulated DE ncRNAs was largely maintained at 1:1, which is consistent with previous studies. The DE ncRNAs identified in this study originated from exons, introns, and intergenic regions, with most DE ncRNAs, especially DE circRNAs, originating from a single exon, which may be related to the current mechanism of circRNA formation in plants: exon skipping events (Conn et al., 2017). In addition, the expression of many circRNAs was extremely low, a feature that may be an essential feature common to circRNAs in plants (Zhang et al., 2020).

In many plant species, ncRNAs have been found to be involved in regulating the expression of their host genes, regulating the physiological and biochemical activities of the plant, and thus regulating the growth life of the plant. In the face of drought, ncRNAs perform different functions to regulate the state of the plant itself. For example, in *Arabidopsis thaliana*, drought-induced lncRNA (DRIR) was confirmed as a novel positive regulator of the plant response to drought and salt stresses (Qin et al., 2017), and miR165/166 use abscisic acid (ABA) signalling to modify plant xylem morphology under conditions of environmental stress (Ramachandran et al., 2018). In cassava, lncRNAs were confirmed to play crucial roles in MT-mediated drought stress responses (Ding et al., 2019). In maize, DRIR represses *ZmNAC111* expression and enhances drought tolerance through RNA-directed DNA methylation (Pang et al., 2019), and several microRNAs can regulate phosphate uptake and affect the growth of primary roots in response to nutrient deficiencies in response to water-deficit stress (Seeve et al., 2019). Therefore, in this study, GO enrichment analysis and KEGG enrichment analysis were performed on host genes of DERNAs to investigate the function of DERNAs in the drought response. GO analysis showed that the host genes were enriched mainly in metabolic process (GO: 0008152), cellular process (GO: 0044763), cell (GO: 0005623), binding (GO: 0005488), and catalytic activity (GO: 0003824). KEGG analysis showed that the most closely related metabolic pathways of the host genes of DERNAs under drought treatment were fatty acid biosynthesis (ko00061), fatty acid metabolism (ko01212), peroxisome (ko04146), fatty acid degradation (ko00071), phenylpropanoid biosynthesis (ko00940), DNA replication (ko03030), starch and sucrose metabolism (ko00500), pentose and glucuronate interconversions (ko00040), plant hormone signal transduction (ko04075), steroid biosynthesis (ko00905) and biosynthesis of various secondary metabolites-Part 2 (ko00998). These pathways are clearly related to the synthesis and degradation of primary and secondary metabolites and involve synergistic interactions between multiple organelles and hormonal signalling. Therefore, we hypothesized that these ncRNAs regulate the functions of various organelles and regulate the levels of various hormones, leading to changes in the metabolism of multiple metabolites and thus resisting the lack of water within the environment.

## Data availability statement

The original contributions presented in the study are publicly available. This data can be found here: NCBI, PRJNA885522 and PRJNA885524.

## Author contributions

XL and YY planned this experiment. YY, YG and YL performed the experiments. YY and YG analyzed the data. YY write this article. All authors contributed to the article and approved the submitted version.

## Funding

This research was funded by the National Key R&D Program of China, 2021YFD2200504_4.

## Conflict of interest

The authors declare that the research was conducted in the absence of any commercial or financial relationships that could be construed as a potential conflict of interest.

## Publisher’s note

All claims expressed in this article are solely those of the authors and do not necessarily represent those of their affiliated organizations, or those of the publisher, the editors and the reviewers. Any product that may be evaluated in this article, or claim that may be made by its manufacturer, is not guaranteed or endorsed by the publisher.
