# Supplementary material for: Identification and differential analysis of noncoding RNAs in response to drought in Phyllostachys aureosulcata f. spectabilis
Source: Front Plant Sci. 2022 Nov 10;13:1040470. doi: 10.3389/fpls.2022.1040470 (PMC9686404; doi:10.3389/fpls.2022.1040470)
Supplement: Supplementary file 1 [file DataSheet_1.docx]

Supplementary Material

# Supplementary Figures and Tables

## Supplementary Tables

Supplementary Table 1. Quality of the sequence data.

Supplementary Table 2. The primers used in this study.

Supplementary Table 3. GO enrichment of RNAs host gene.

Supplementary Table 4. KEGG enrichment of RNAs host gene.

Supplementary table 5. All DE ncRNAs among all treatments.

Supplementary Table 6. circRNA target

Supplementary Table 7. sRNA target

Supplementary Table 8. DE lncRNA target-A

Supplementary Table 8. DE lncRNA target-B

Supplementary Table 8. DE lncRNA target-C

Supplementary Table 8. DE lncRNA target-D

Supplementary Table 8. DE lncRNA target-E

## Supplementary Figures


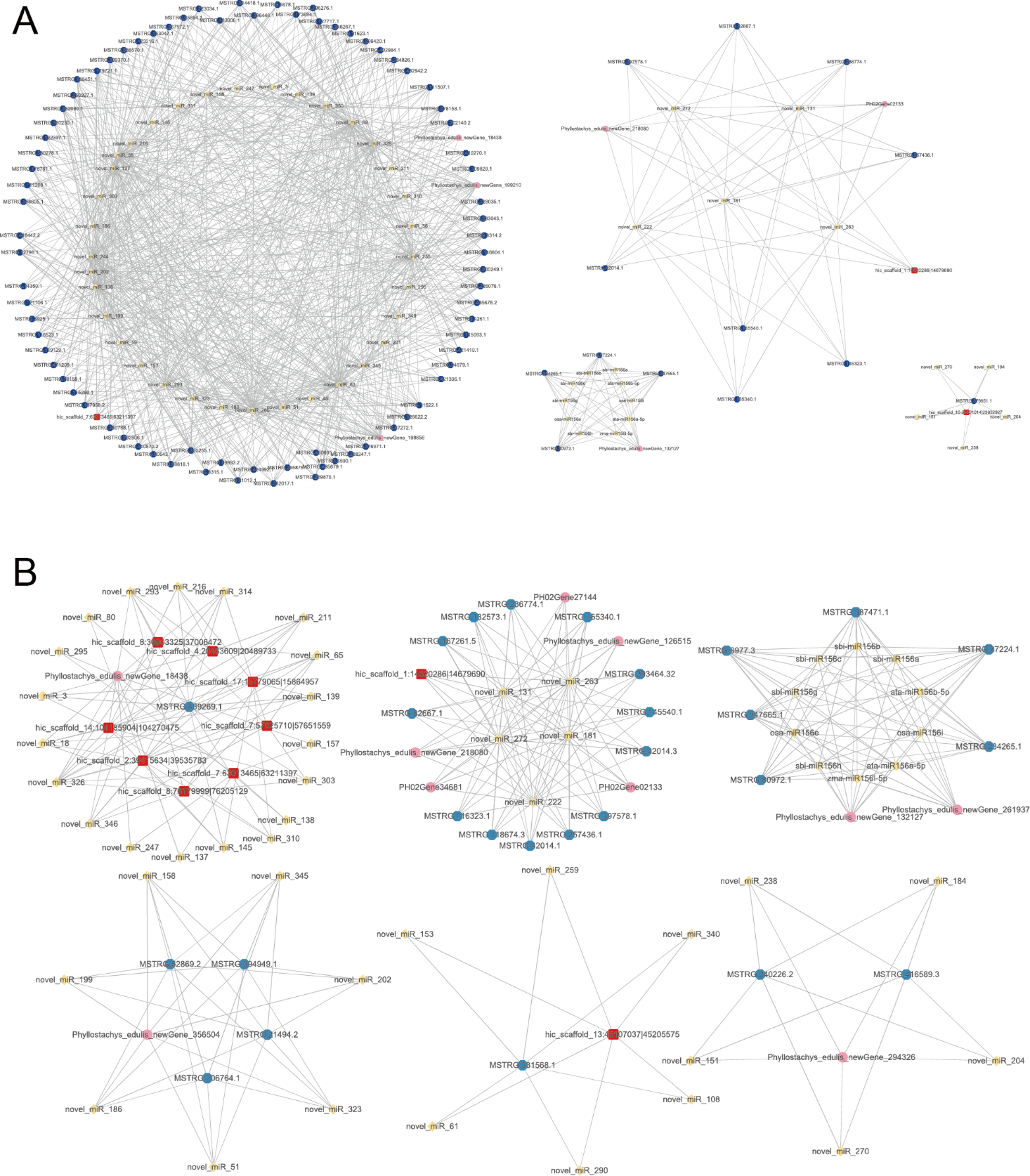


**Supplementary Figure 1.** The mRNA-miRNA-circRNA-lncRNA interaction network. Yellow nodes: miRNAs. Red nodes: circRNAs that may be miRNA decoys. Blue nodes: lncRNAs that may be miRNA decoys. Pink nodes: mRNAs that may be miRNA targets. (A) The reciprocal relationship networks of P1 treatment with drought treatment P2. (B) The reciprocal relationship networks of P1 treatment with drought treatment P3.
